# Supplementary material for: Impact of the COVID-19 Pandemic on Antibiotic Prescribing by Dentists in Galicia, Spain: A Quasi-Experimental Approach
Source: Antibiotics (Basel). 2022 Jul 29;11(8):1018. doi: 10.3390/antibiotics11081018 (PMC9404831; doi:10.3390/antibiotics11081018)
Supplement: Supplementary file 1 [file antibiotics-11-01018-s001.zip › antibiotics-1823555-supplementary.pdf]

Appendix A

Table S1. Defined daily dose by active ingredient as prescribed by primary-care dentists, according to the AWaRe Classification[49].

Antibiotic	ATC code	Category	DDD WHO *	DDD prescribed
Amoxicillin	J01CA04	Access	1.5	899795
Amoxicillin/clavulanic acid	J01CR02	Access	1.5	791368
Azithromycin	J01FA10	Watch	0.3	62668
Clindamycin	J01FF01	Access	1.2	38034
Cefuroxime	J01DC02	Watch	0.5	13906
Erythromycin	J01FA01	Watch	1.0	11203
Spiramycin	J01FA02	Watch	3.0	7992
Miocamycin	J01FA11	Watch	1.2	3009
Clarithromycin	J01FA09	Watch	0.5	2863
Ciprofloxacin	J01MA02	Watch	1.0	2377
Levofloxacin	J01MA12	Watch	0.5	1631
Doxycycline	J01AA02	Access	0.1	1126
Cefixime	J01DD08	Watch	0.4	338
Moxifloxacin	J01MA14	Watch	0.4	333
Cloxacilin	J01CF02	Access	2.0	285
Phenoxymethylpenicillin	J01CE02	Access	2.0	268
Josamycin	J01FA07	Watch	2.0	228
Cefditoren	J01DD16	Watch	0.4	270
Fosfomycin	J01XX01	Watch	3.0	223
Cefalexin	J01DB01	Access	2.0	210
Minocycline	J01AA08	Watch	0.2	132
Sulfamethoxazole/trimethoprim	J01EC01	Access	2.0	110
Norfloxacin	J01MA06	Watch	0.8	35
Procaine-benzylpenicillin	J01CE09	Access	0.6	28
Nitrofurantoin	J01XE01	Access	0.2	21
Benzylpenicillin	J01CE01	Access	3.6	20
Cefaclor	J01DC04	Watch	1.0	20
Roxithromycin	J01FA06	Watch	0.3	20
Benzathine-benzylpenicillin	J01CE08	Access	3.6	17
Ampicillin	J01CA01	Access	2.0	15

DDD=Defined daily dose

*** DDD defined by WHO index 2022**
